# Structural and functional characterization of deep-sea thermophilic bacteriophage GVE2 HNH endonuclease

**DOI:** 10.1038/srep42542

**Published:** 2017-02-13

**Authors:** Likui Zhang, Dandan Xu, Yanchao Huang, Xinyuan Zhu, Mianwen Rui, Ting Wan, Xin Zheng, Yulong Shen, Xiangdong Chen, Kesen Ma, Yong Gong

**Affiliations:** 1 Marine Science & Technology Institute Department of Environmental Science and Engineering, Yangzhou University, China; 2 Institute of Health Sciences and School of Life Science, Anhui University, Hefei, Anhui 230601, China; 3 Beijing Synchrotron Radiation Facility, Institute of High Energy Physics, Chinese Academy of Sciences, China; 4 State Key Laboratory of Microbial Technology, Shandong University, China; 5 State Key Laboratory of Virology, College of Life Sciences, Wuhan University, Wuhan 430072, China; 6 Department of Biology, University of Waterloo, Canada

## Abstract

HNH endonucleases in bacteriophages play a variety of roles in the phage lifecycle as key components of phage DNA packaging machines. The deep-sea thermophilic bacteriophage *Geobacillus* virus E2 (GVE2) encodes an HNH endonuclease (GVE2 HNHE). Here, the crystal structure of GVE2 HNHE is reported. This is the first structural study of a thermostable HNH endonuclease from a thermophilic bacteriophage. Structural comparison reveals that GVE2 HNHE possesses a typical ββα-metal fold and Zn-finger motif similar to those of HNH endonucleases from other bacteriophages, apart from containing an extra α-helix, suggesting conservation of these enzymes among bacteriophages. Biochemical analysis suggests that the alanine substitutions of the conserved residues (H93, N109 and H118) in the HNH motif of GVE2 HNHE abolished 94%, 60% and 83% of nicking activity, respectively. Compared to the wild type enzyme, the H93A mutant displayed almost the same conformation while the N108A and H118A mutants had different conformations. In addition, the wild type enzyme was more thermostable than the mutants. In the presence of Mn^2+^ or Zn^2+^, the wild type enzyme displayed distinct DNA nicking patterns. However, high Mn^2+^ concentrations were needed for the N109A and H118A mutants to nick DNA while Zn^2+^ inactivated their nicking activity.

Most HNH endonucleases that can nick double-stranded DNA sites ranging from 3 to 5 bp in the presence of a divalent metal ion contain a conserved catalytic HNH motif and a zinc-binding site [CxxC]_2_^1^. HNH endonucleases are present in many bacteriophages and prophages. The location of an HNH endonuclease gene in phage genomes is next to a terminase gene and is highly conserved, suggesting a possible biological role in the stimulation of homologous recombination by nicking DNA, which further enhances gene conversion. Thus, HNH endonucleases in phages play important roles in the phage lifecycle as key components of phage DNA packaging machines^2^,^3^.

HNH endonucleases have large group members from various organisms, including HEases (homing endonuclease), REases (restriction endonuclease), structure-specific endonucleases, non-specific nucleases, CRISPR (clustered regularly interspaced short palindromic repeat)-associated protein Cas9 and DNA repair enzymes^4^. Biochemical and structural studies have provided a wealth of molecular details on HNH endonucleases of bacteria and bacteriophages. Structures are now available for ColE7, ColE9, I-*Hmu*I, *Pac*I, *Hpy*99I, and *Geobacter metallireducens* GS-15 HNH endonuclease (Gme HNHE)^5^,^6^,^7^,^8^,^9^,^10^,^11^. Most HNH endonucleases adopt a similar structure, comprising two antiparallel β-strands, an α-helix and a divalent metal ion bound in the active center. Thus, the HNH motif in HNH endonucleases is referred to as a ββα-metal fold. The active site of HNH endonucleases consists of two highly conserved His and Asn, and a variable His (Asn in second superfamily HNH endonucleases). The first conserved His in the HNH motif is located at the end of the first β-strand (Fig. 1A), and serves as the general base to activate the water molecule, which attacks the DNA backbone^12^. The second conserved Asn in the HNH motif plays an important role in positioning of the two β-strands correctly^12^. Furthermore, the third His, Lys or Asn in the HNH motif is located at the conserved α-helix, and is thought to participate in metal binding^13^. Several studies show that the active site in HNH endonucleases has been found in site-specific homing endonucleases^14^, colicins^15^, soluble pyocins^16^, restriction enzymes^17^ and bacterial factors involved in developmentally controlled DNA rearrangements^18^, suggesting that these enzymes are evolutionarily related and employ a similar catalytic mechanism.

However, much less is known about HNH endonucleases in thermophilic bacteriophages. No structures are currently available for any thermostable HNH endonuclease. An HNH endonuclease from the thermophilic bacteriophage GVE2, a thermophilic and lytic bacteriophage that infects *Geobacillus* sp. E263 isolated from a deep-sea hydrothermal field in the east Pacific^19^,^20^, was the first thermostable member of HNH endonucleases from thermophilic bacteriophages to be biochemically characterized^21^. Xu *et al*. reported the nicking sites of GVE2 HNHE in the presence of Mg^2+^ 4. Sequence analysis suggests that GVE2 HNHE possesses an active site similar to those in other HNH endonucleases^21^. Our previous studies suggest that the residue H93 in GVE2 HNHE is a critical residue in the active site for nicking DNA^4^.

Here, we reported the crystal structures of GVE2 HNHE and investigated the function of the key residues in the HNH motif of the enzyme for the first time. We show that GVE2 HNHE possesses a conserved ββα-metal fold with those of HNH endonucleases from other bacteriophages, demonstrating that there is a common evolutionary origin of these enzymes in bacteriophages. Importantly, GVE2 HNHE displays different conformational structure from other HNH endonucleases, which may be the basis for the enzyme to nick DNA at high temperature. In addition, GVE2 HNHE nicks DNA with distinct patterns in the presence of Mn^2+^ or Zn^2+^.

## Results

### Overall structure of GVE2 HNHE

**Figure 1:**
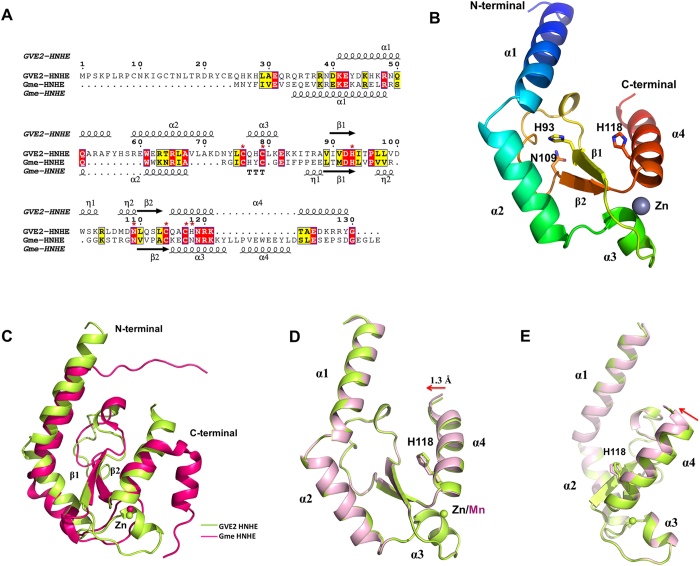
The crystal structures of GVE2 HNHE. (**A**) Sequence alignment of GVE2 HNHE and Gme HNHE. Strictly conserved and conservatively substituted residues are marked with red and yellow backgrounds. The secondary structure elements at the top and bottom are from our structure and Gme HNHE (PDB: 4H9D), respectively. The conserved amino acid residues in HNH motif and [CxxC]_2_ motif are indicated with a red “*”. (**B**) The overall structure of GVE2 HNHE. GVE2 HNHE possesses 4 α-helixes and two β-sheets. The Zn ion is shown as a grey sphere. Labeled residues in sticks indicate H93, N109 and H118. (**C**) The structural comparison of GVE2 HNHE (green) and Gme HNHE (red). The structure of GVE2 HNHE is superimposed well with that of Gme HNHE. GVE2 HNHE contains an extra α-helix (α3) and has different length of the conserved α-helix (α4). (**D**) Structural comparison of GVE2 HNHE with Zn^2+^ (green) and Mn^2+^ (pink). The two structures are highly similar, except the C-terminus of helix α4 shifts by a distance of approximately 1.3 Å upon binding of Mn^2+^ ion, as red arrows indicated. Residues H118 are shown as sticks. (**E**) View of (D) rotated around the vertical axis by 90°.

The crystal structure of GVE2 HNHE was determined at 1.52 Å resolution using the SAD (single-wavelength anomalous diffraction) method to calculate the initial phases (data collection and refinement statistics are shown in Table 1, and the protein sequence is shown in Fig. 1A). Note that we solved the crystal structure of the truncated GVE2 HNHE from N39 to G130. The initial 38 residues (M1-R38) could not be solved because of the disordered electron density, indicating the N-terminal region of the protein is very flexible. The overall structure of GVE2 HNHE contains two antiparallel β-sheets and four α-helices, and shares a conserved ββα-metal fold with those of other HNH endonucleases (Fig. 1B). Like other endonucleases, a zinc ion exists in the center of the structure of GVE2 HNHE and is coordinated by Cys76 (in the short loop linking helix α2 and α3), Cys79 (in the helix α3), Cys114 (in the short loop linking the helix α4 and β2), and Cys117 (in the helix α4 at the end of the C-terminal domain). It resembles the Zn^2+^-bound structure of Gme HNHE^11^, suggesting that the Zn-finger domain in HNH endonuclease is conserved.

### Comparison of GVE2 HNHE and its homologous proteins

Based on the DALI search (http://ekhidna.biocenter.helsinki.fi/dali_server), structural homologs of GVE2 HNHE included the following HNH endonucleases: Gme HNHE from *Geobacter metallireducens* GS-15 (PDB: 4H9D)^11^, periplasmic nuclease Vvn from *Vibrio vulnificus* (PDB: 1OUO)^22^, the Type II Cas9 endonuclease from *Actinomyces naeslundii* (PDB: 4OGC)^23^, and endonuclease I from *Vibriosalmonicida* (PDB: 2PU3)^24^, which share 15–24% amino acid sequence identity with GVE2 HNHE.

**Figure 2:**
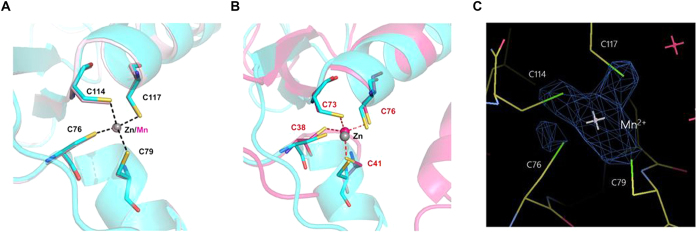
The Zn-finger domain of GVE2 HNHE. (**A**) The Zn^2+^-binding sites of GVE2 HNHE (blue). Residues C76, C79, C114 and C117, which coordinate with Zn^2+^, are shown as sticks. Zinc ion is shown as a grey sphere. It is superimposed with Mn^2+^-bound structure (pink). (**B**) Superimpositions of the Zn^2+^-binding sites of GVE2 HNHE (blue) and Gme HNHE (Red). Zinc ion and residues involved in zinc ions coordination of Gme HNHE are labeled. (**C**) The anomalous difference Fourier map (blue, contour at 4σ) shown for Mn^2+^ ion-bound in GVE2 HNHE.

Matched structure superimposition was found between GVE2 HNHE and Gme HNHE (PDB: 4H9D) with Cα atom r.m.s.d of 3.2 Å and Z scores of 2.7. Sequence alignment of GVE2 HNHE and Gme HNHE and their structural comparison are shown in Fig. 1A,C. Although both HNH endonucleases contain the zinc ion and the conserved catalytic residues in the HNH motif (Figs 1C and 2B), the structural discrepancies occur between GVE2 HNHE and Gme HNHE. Firstly, GVE2 HNHE possesses an extra α-helix (α3: residues 85–92) at the C-terminus, which is absent in Gme HNHE. At the same time, GVE2 HNHE lacks the last α-helix, which is present in the C-terminus of Gme HNHE. Furthermore, α1-, α2-, and α4-helixes in GVE2 HNHE display differences in position and angle with corresponding α1-, α2- and α3-helixes in Gme HNHE (Fig. 1C). These structural differences may enable GVE2 HNHE and Gme HNHE to nick DNA in different environments.

### The crystal structure of GVE2 HNHE with Mn^2+^

Our previous studies suggest that Mn^2+^ is the optimal divalent metal ion for GVE2 HNHE to nick DNA^21^. To reveal how the enzyme associates with Mn^2+^, we solved the structure of GVE2 HNHE in the presence of Mn^2+^ at 1.53 Å resolution (Fig. 1D). The structure of GVE2 HNHE with Mn^2+^ shows that a manganese ion associates with the conserved [CxxC]_2_ motif (Fig. 2A), which resembles that of the enzyme with Zn^2+^. Based on the anomalous difference Fourier map calculated from the data collected at the absorption edge of Mn^2+^ ion (λ = 1.8 Å) (Fig. 2C), we deduced that the Mn^2+^ ion was bound at the same site with the Zn^2+^ ion in our Mn^2+^-bound GVE2 HNHE structure, but we still cannot elude the possibility that the binding site is simultaneously occupied by both Mn^2+^ and Zn^2+^.

There is no significant structural change for the two structures of GVE2 HNHE with Mn^2+^ and Zn^2+^, however, the C-terminus of GVE2 HNHE shifts by a distance of approximately 1.3 Å upon binding of Mn^2+^ (Fig. 1D). The coordination bond lengths formed between the manganese ion and the conserved residues in the [CxxC]_2_ motif in GVE2 HNHE is slightly different with these of the zinc ion (Fig. 2A), which further induces the position of α4-helix in the structure of the enzyme to swing around by approximately 5°. In addition, the conformations of residue H118 on α4-helix are different in two structures (Fig. 1E). The overall structure change of GVE2 HNHE in the presence of Mn^2+^ may enable the enzyme to have the optimal DNA nicking activity. The structural difference of GVE2 HNHE in the presence of Mn^2+^ or Zn^2+^ would provide the basis for nicking DNA in distinct patterns (see below).

**Figure 3:**
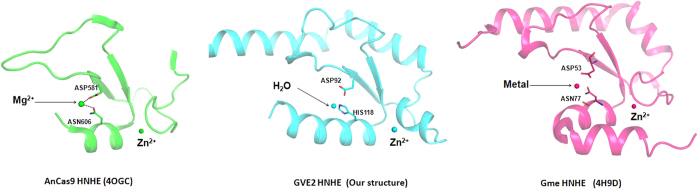
Comparison of the crystal structure of HNH motif of GVE2 HNHE, AnaCas9 HNHE and Gme HNHE. The crystal structure of AnaCas9 HNHE and Gme HNHE was modified by Pymol software. AnaCas9 HNHE and Gme HNHE contain a conserved Asn (Asn606 in AnaCas9 HNHE and Asn77 in Gme HNHE) that associates with an additional metal ion, whereas GVE2 HNHE harbors a His at this position.

Structural studies suggest that HNH endonucleases with a Zn-finger motif may coordinate an additional divalent metal ion in their HNH motifs except for Zn ion^25^. For example, an additional Mg^2+^ is coordinated by associating with the conserved residues Asp581 and Asn606 in the HNH motif of AnaCas9 HNHE (Fig. 3). Although it remains unknown, an additional metal ion is also coordinated by the conserved residues Asp53 and Asn77 in the HNH motif of Gme HNHE (Fig. 3). However, no additional metal ion was observed in the HNH motif of GVE2 HNHE (Fig. 3). Comparison of these three structures of AnaCas9 HNHE, Gme HNHE and GVE2 HNHE suggests that Asn is conserved in this position in both the AnCas9 HNHE and Gme HNHE, but His is found in GVE2 HNHE (Fig. 3); thus, a His at this position may lead to no binding of an additional metal ion in GVE2 HNHE. Although no binding of additional metal ion was observed in GVE2 HNHE, we cannot rule out the possibility that an additional metal ion may be bound into the active site of GVE2 HNHE after the enzyme binds to DNA substrate.

### The active sites of GVE2 HNHE

HNH endonucleases possess a highly conserved ββα-metal fold motif, which is thought to play an important role in nicking DNA. Sequence analysis shows that residues H93, N109 and H118 in GVE2 HNHE are the key residues in the conserved ββα-metal fold motif, and could be components of the active site of the enzyme (Fig. 1B). Residue H93 in GVE2 HNHE is at the end of the strand β1 of the ββα-metal fold region on the surface of the structure, which is equivalent to the first ‘H’ of the HNH motif. Residue N109 in GVE2 HNHE is located at initiation site of strand β2, which is the second ‘N’ of the HNH motif. Residue H118 in GVE2 HNHE lies in the center of the α4-helix at the end of the C-terminal, which is equivalent to the third ‘H’ of the HNH motif. Our previous study revealed that the GVE2 HNHE H93A mutant abolishes DNA nicking activity^21^, supporting the idea that the residue H93 is one of key residues in the active site center of the enzyme.

### Conformational change caused by GVE2 HNHE mutants

**Figure 4:**
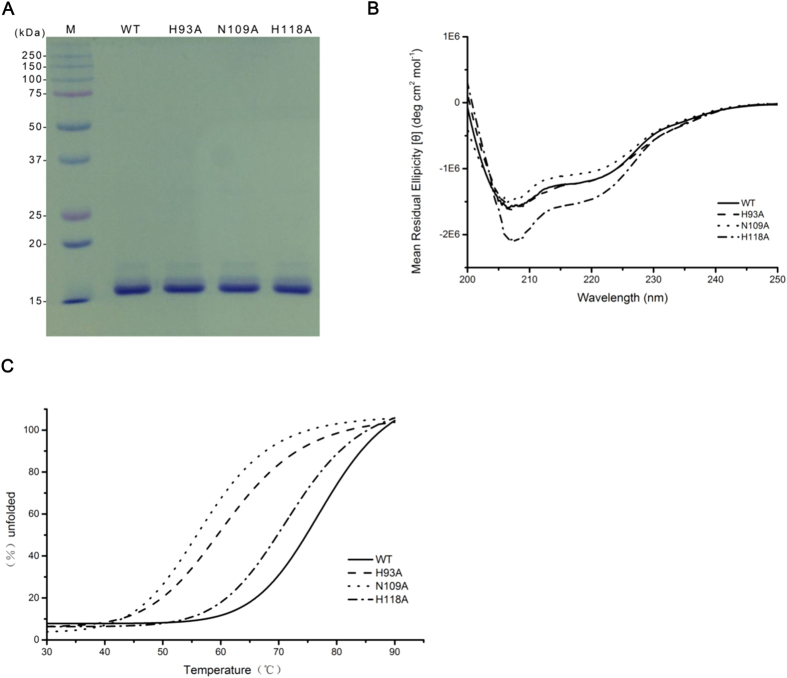
Purification, conformation change, and thermostability of the wild type and mutant GVE2 HNHEs. (**A**) SDS-PAGE of the purified GVE2 HNHE and its H93A, N109A and H118A mutants. (**B**) Secondary structure assays of the wild type and mutant GVE2 HNHEs by CD analysis. Changes in secondary structure were monitored by scanning from 200 to 250 nm and the mean residue ellipticity was recorded with different line patterns for the wild type and mutant proteins as indicated. (**C**) Thermal stability assay of the wild type and mutant GVE2 HNHEs by CD analysis. The thermal unfolding of the proteins was determined at 222 nm as described under “Methods”. The melting curves of the wild type and mutant proteins are shown in different line patterns as indicated.

As discussed above, residues H93, N109 and H118 in GVE2 HNHE are key amino acid residues for DNA nicking. To discern the biochemical function of the conserved HNH motif in GVE2 HNHE, we constructed another two mutants of the enzyme: N109A and H118A. Note that the H93A mutant was constructed in our previous work^21^. The purified wild type and mutant GVE2 HNHEs are shown in Fig. 4A.

To determine whether the H93A, N109A or H118A mutants cause the overall structure change of GVE2 HNHE, we performed the CD (circular dichroism) analysis of the wild type and mutant enzymes. The H93A mutant displayed almost the same structure as the wild type enzyme (Fig. 4B), suggesting that the H93A substitution did not change the overall structure of the enzyme. However, the N109A and the H118A mutants displayed different conformational change from the wild type enzyme, indicating that the N109A and the H118A substitutions disrupted the overall structure of the enzyme.

### Reduced thermostability of GVE2 HNHE mutants

To examine whether the substitutions of H93A, N109A, and H118A affect thermostability of GVE2 HNHE, we performed thermal unfolding assays by CD analysis using the wild type and mutant enzymes. The thermal unfolding curves revealed that the wild type GVE2 HNHE is more thermostable than the H118A, H93A, N109A mutants (Fig. 4C), suggesting that the H118A, H93A, N109A substitutions decreased the thermostability of the enzyme in varying degrees.

### Reduced DNA nicking of GVE2 HNHE mutants

**Figure 5:**
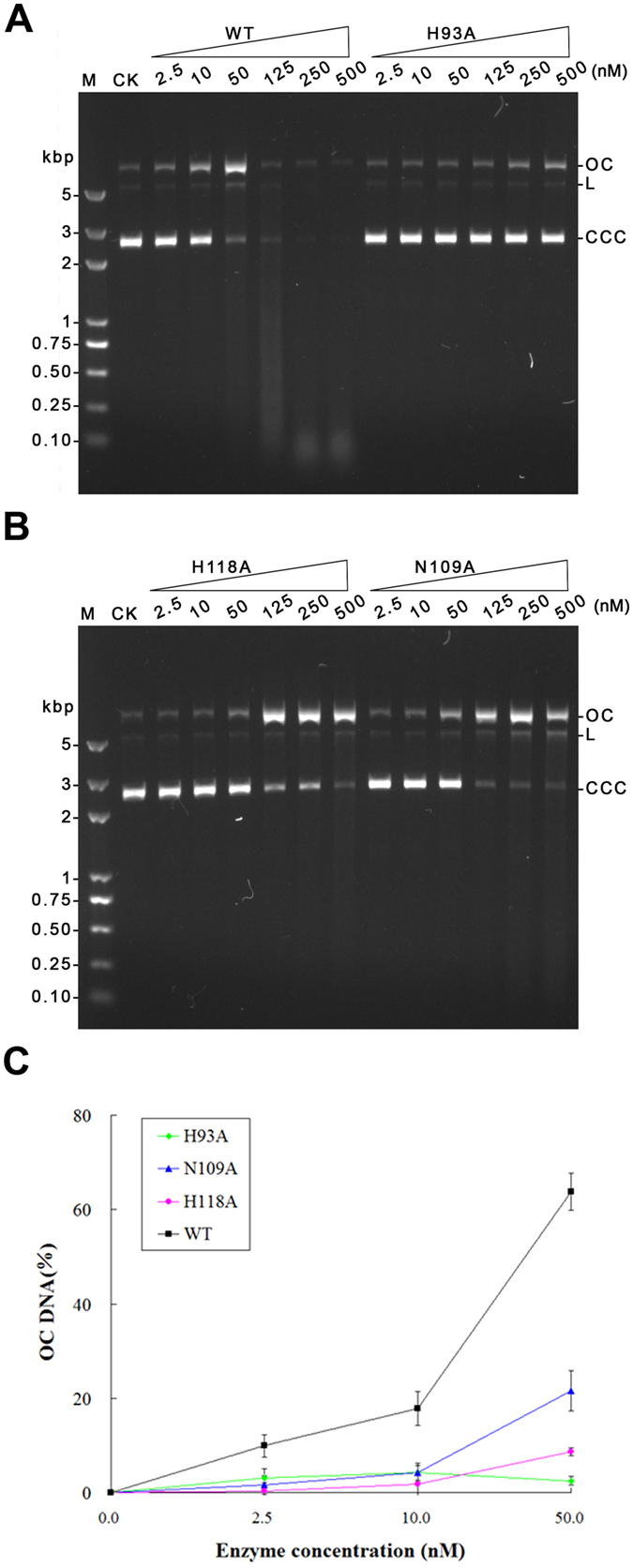
DNA nicking assays of the wild type and mutant GVE2 HNHEs. (**A**) DNA nicking by the wild type and H93A mutant enzymes. DNA nicking assays of various concentrations (2.5, 10, 50, 125, 250 and 500 nM) of GVE2 HNHE and its H93A mutant were performed in the presence of 2 mM Mn^2+^ by using pET-30a DNA as substrates at 60 °C for 15 min. (**B**) DNA nicking by the N109A and H118A mutants. DNA nicking assays were performed by the N109A and H118A mutants as the same in (**A**). (**C**) Comparison of nicking efficiency of the wild type and mutant GVE2 HNHEs. DNA nicking reactions were carried out by the wild type and mutant GVE2 HNHEs (2.5, 10 and 50 nM). The ocDNA product was quantified. CK: the reaction without the enzyme; OC: open circular DNA; L: Linear DNA; CCC: covalently closed circular DNA.

To determine whether the H93A, N109A and H118A GVE2 HNHE mutants nick DNA, we used the plasmid DNA as substrate and Mn^2+^ as a cofactor because the enzyme is a DNA nicking enzyme and Mn^2+^ is the optimal divalent metal ion for DNA nicking^21^. Compared to the wild type enzyme, the H93A mutant displayed undetectable DNA nicking activity even in the presence of 500 nM of enzyme concentration (Fig. 5A), which is consistent with our previous study^21^. However, the N109A and H118A mutants were capable of nicking DNA when the mutants with high concentrations ranging from 125 to 500 nM were employed in the DNA nicking reactions. When a relatively lower concentration (50 nM) of the wile type or mutant enzyme was used in the DNA nicking reaction, the N109A and H118A mutants had slightly detectable nicking activity while the wild type enzyme displayed clear DNA nicking activity (Fig. 5B). By quantifying the ocDNA product created by the H93A, N109A and H118A GVE2 HNHE mutants, we found that the H93A, N109A and H118A substitutions in GVE2 HNHE caused 94%, 60% and 83% loss of activity of the enzyme (Fig. 5C), respectively. These observations suggest that residues of H93, N109 and H118 in GVE2 HNHE are critical for the DNA nicking of the enzyme.

### The effect of Mn^2+^ on DNA nicking of the wild type and mutant GVE2 HNHEs

**Figure 6:**
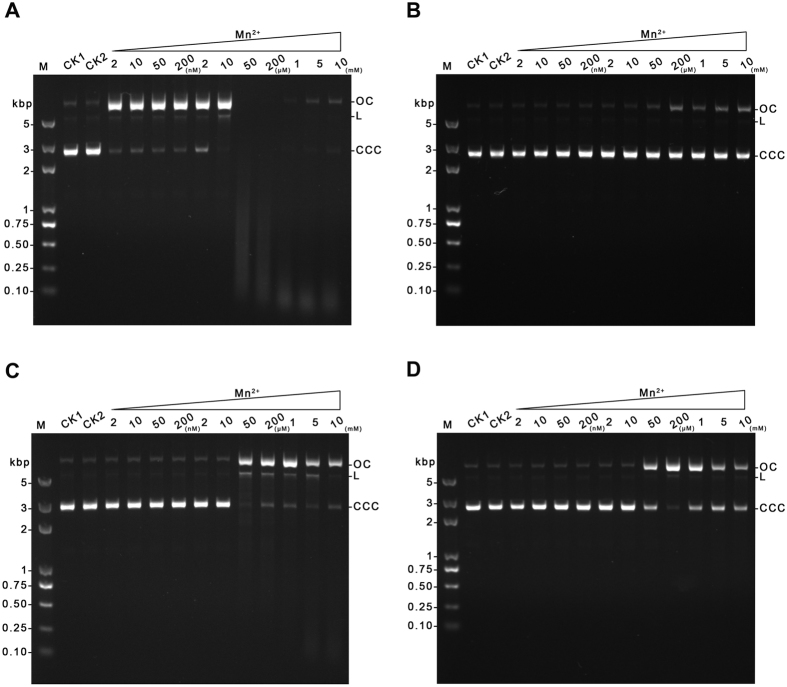
Effect of various Mn^2+^ concentrations on the DNA nicking of the wild type and mutant GVE2 HNHEs. (**A**) DNA nicking of the wild type GVE2 HNHE. (**B**) DNA nicking of the H93A mutant. (**C**) DNA nicking of the N109A mutant. (**D**) DNA nicking assays of the H119A mutant. DNA nicking reactions were performed at various reaction Mn^2+^ concentrations ranging from 0.002 to 10 mM by using pET-30a DNA as the substrate at 60 °C for 15 min. CK1: the reaction without the enzyme. CK2: the reaction without Mn^2+^. OC: open circular DNA; L: Linear DNA; CCC: covalently closed circular DNA.

In our previous work, we demonstrated that Mn^2+^ is the optimal divalent ion for DNA nicking by GVE2 HNHE only when the reactions are performed in the presence of 0.2–2 mM Mn^2+^ 21. To investigate the effect of Mn^2+^ on the wild type and mutant GVE2 HNHEs, we performed DNA nicking reactions in the presence of a broad range of Mn^2+^ concentrations from 2 nM to 10 mM. We found that the wild type GVE2 HNHE cleaved the cccDNA (covalently closed circular DNA) substrate to form the ocDNA (open circular DNA) product even in the presence of very low Mn^2+^ (2 nM) (Fig. 6A). When Mn^2+^ concentrations were increased to 10 μM, only ocDNA product was created by the wild type GVE2 HNHE, suggesting that 2 nM to 10 μM Mn^2+^ in the DNA nicking reactions enabled the enzyme to transform the cccDNA substrate to the ocDNA product. In addition, when Mn^2+^ concentrations were increased to more than 50 μM, smear DNA product and small linear DNA fragments were formed in DNA nicking reaction catalyzed by the wild type GVE2 HNHE. These observations suggest various concentrations of Mn^2+^ can stimulate GVE2 HNHE to nick the cccDNA substrate into various DNA products.

Compared to the wild type enzyme, the H93A mutant was not able to nick the cccDNA substrate to form any DNA product in the presence of Mn^2+^ ranging from 2 nM to 10 mM (Fig. 6B), which is similar with our previous study^21^. In addition, the low concentration of Mn^2+^ (less than 10 μM) did not enable the N109A and H118A mutants to nick DNA. However, the N109A and H118A mutants nicked the cccDNA substrate into the ocDNA product when Mn^2+^ concentrations ranging from 50 μM to 10 mM were used in the DNA nicking reactions (Fig. 6C,D). Therefore, these observations suggest that residue H93 in GVE2 HNH endonuclease is a key residue for DNA nicking, and N108 and H118A residues may play two important roles in maintaining structural conformation and nicking DNA.

### The effect of Zn^2+^ on DNA nicking of the wild type and mutant GVE2 HNHEs

**Figure 7:**
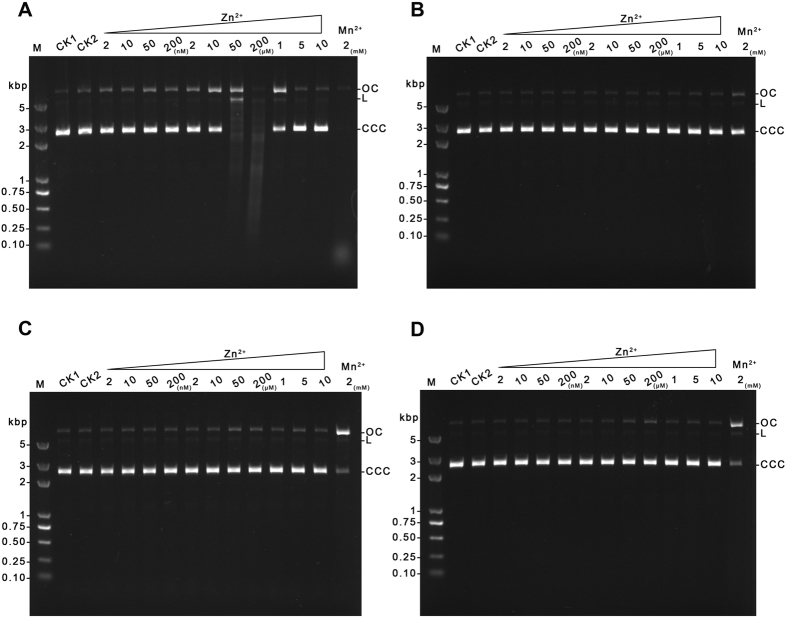
Effect of various Zn^2+^ concentrations on the DNA nicking of the wild type and mutant GVE2 HNHEs. (**A**) DNA nicking of the wild type GVE2 HNHE. (**B**) DNA nicking of the H93A mutant. (**C**) DNA nicking of the N109A mutant. (**D**) DNA nicking of the H119A mutant.DNA nicking reactions were performed at various reaction Zn^2+^ concentrations ranging from 0.002 to 10 mM by using pET-30a DNA as the substrate at 60 °C for 15 min. CK1: the reaction without the enzyme. CK2: the reaction without Zn^2+^. Mn^2+^: the reaction with 2 mM Mn^2+^. OC: open circular DNA; L: Linear DNA; CCC: covalently closed circular DNA.

Biochemical and structural analyses suggest that a Zn-finger motif is another important motif in most HNH endonucleases. Our previous work revealed that Zn^2+^ can stimulate GVE2 HNHE to nick DNA^21^. To investigate the effect of Zn^2+^ on DNA nicking activity of the wild type and mutant GVE2 HNHEs, we performed DNA nicking assays in the presence of 2 nM to 10 mM Zn^2+^. We found that Zn^2+^ enabled the wild type GVE2 HNHE to nick DNA only in the presence of 10 μM to 1 mM. Both high Zn^2+^ concentration (more than 10 mM) and low Zn^2+^ concentration (less than 10 μM) inhibited the wild type GVE2 HNHE to nick DNA (Fig. 7A). Thus, GVE2 HNHE displayed different DNA nicking patterns when using Zn^2+^ or Mn^2+^ as a cofactor. Interestingly, we found that the H93A, N109A and H118A mutants abolished the DNA nicking activity regardless of what Zn^2+^ concentrations were employed in the DNA nicking reactions (Fig. 7B–D), which contrasts sharply to the DNA nicking in the presence of Mn^2+^ where high concentrations of Mn^2+^ enable the mutants to nick DNA. These observations suggest that Zn^2+^ plays important roles in both maintaining the structural conformation of GVE2 HNHE and nicking DNA.

### Mn^2+^- and Zn^2+^-binding of GVE2 HNHE

**Figure 8:**
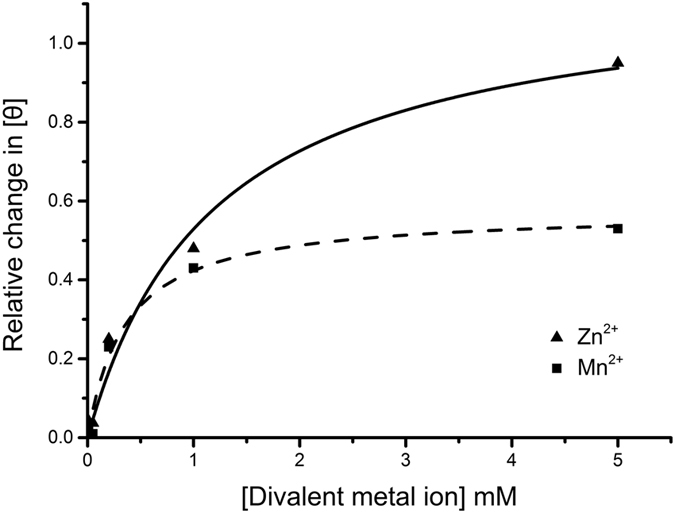
Mn^2+^- and Zn2^+^-binding of GVE2 HNHE. The relative changes in the mean residue ellipticity were monitored at 222 nm as described under “Methods”. The hyperbolic curves were plotted by Origin software. The *K*_d_ values for Mn^2+^- and Zn^2+^-binding of GVE2 HNHE were calculated to be 0.36 ± 0.08 mM and 1.29 ± 0.29 mM, respectively.

To reveal the mechanistic process of DNA nicking by GVE2 HNHE in the presence of Mn^2+^ or Zn^2+^, we performed the Mn^2+^- and Zn^2+^-binding of the enzyme by CD analysis, respectively. Based on the plotting curves, we found that the *K*_d_ values for GVE2 HNNE to bind to Mn^2+^ and Zn^2+^ are 0.36 ± 0.08 mM and 1.29 ± 0.29 mM (Fig. 8), respectively. This observation suggests that GVE2 HNHE has a higher affinity for binding to Mn^2+^ than to Zn^2+^, which is consistent with the above result that Mn^2+^ is better than Zn^2+^ for enabling the enzyme to nick DNA.

## Discussion

The present study provided structural and biochemical mechanistic insight into the function of HNH motif of the thermostable GVE2 HNHE for the first time. The crystal structure shows that GVE2 HNHE possesses a conserved HNH motif and a Zn-finger motif, which are widespread in HNH endonucleases. Furthermore, a conserved ββα-metal fold in HNH endonucleases is also observed in the crystal structure of GVE2 HNHE, suggesting that the enzyme shares a similar conformation with other HNH endonucleases. However, GVE2 HNHE has several unique structural characteristics, compared with other HNH endonucleases. Firstly, GVE2 HNHE possesses an extra α-helix that is located at the initial part of the first β-sheet strand. We proposed that the extra α-helix in GVE2 HNHE would be helpful for maintaining structural conformation, which may further enable the enzyme to nick DNA at high temperature. Our proposal is being investigated in our laboratory by engineering of the GVE2 HNHE mutant that lacks the extra α-helix. Secondly, GVE2 HNHE has different lengths of α-helix, especially for the conserved α-helix in the ββα-metal fold, forming the specifically structural conformation of the enzyme. In addition, the ββα-metal fold in GVE2 HNHE possesses several amino acid residues, which are distinct from those of other HNH endonucleases. Overall, these structural differences in GVE2 HNHE may provide a basis for its nicking DNA at high temperature.

Previous studies suggest that three conserved residues in the HNH motif play an important role in DNA nicking in the other HNH endonucleases^26^,^27^,^28^,^29^. Herein, we found that the residue H93 in the HNH motif in GVE2 HNHE is a key residue for DNA nicking because the substitution from H to A enabled the enzyme to abolish the ability to nick DNA (Fig. 5A). Similarly, the DNA hydrolysis activities of the H545A N-ColE7 mutant and the H116A EheA mutant were completely abolished^26^,^27^, where EheA is a thermostable HNH endonuclease from the bacterium *Exiguobacterium* sp. yc3. However, CD analysis results showed that the H93A substitution did not cause overall structural change of GVE2 HNHE (Fig. 4B) while the substitution affected the fold of GVE2 HNHE (Fig. 4C), which may lead to a reduction in nicking DNA. Thus, residue H93 in GVE2 HNHE is important for DNA nicking.

Residues N109 and H118 in GVE2 HNHE are another two conserved residues in the HNH motif of HNH endonucleases. The N109A and H118A mutants retained about 50~80% DNA nicking activity of the wild type GVE2 enzyme, which is in sharp contrast to the same observations in EheA N141 and N156 mutants^26^ while resembles the N560 and H573 mutants that partially abolished 6.9% to 83.2% of the wild type enzyme activity^28^. The substitutions in the residues H116, N141 and N156 mutants resulted in the complete loss of DNA nicking activity of the wild type EheA^26^. These differences in the active sites between GVE2 HNHE and EheA may be due to various structures of the two enzymes and various origins. In addition, these substitutions of N109A and H118A disrupted the overall conformational change of the wild type enzymes (Fig. 4B), and also affected the folds of the wild type enzymes, suggesting that residues N109 and H118 are essential for both maintaining their structural roles and nicking DNA.

Our previous work suggested that GVE2 HNHE is dependent on a divalent metal ion and Mn^2+^ is optimal for its nicking DNA^21^. A few HNH endonucleases from bacteriophages, including *Lactobacillus* phage Lrm1 gp54 (N.ϕLrm1), *S. aureus* prophage ORF (open reading frame) Sap040a_009 HNHE, N.BceSVIII, phage N.φGamma, and Gp54 of *Lactobacillus* phage Lrm1 (N.fLrm1) are active in nicking DNA in the presence of Mn^2+^ ranging from 1–10 mM^4^. In addition, Vasu *et al*. suggested that the D148G mutant of *Kpn*I restriction endonuclease is a Mn^2+^-dependent sequence specific endonuclease, defective in DNA cleavage with Mg^2+^ and other divalent metal ions^30^. However, GVE2 HNHE prefers a low concentration of Mn^2+^ (from 2 nM to 10 μM) in nicking DNA (Fig. 6A) and its mutants need higher concentrations of Mn^2+^ to nick DNA (Fig. 6B–D). Therefore, the necessity for the optimal Mn^2+^ concentration supporting maximum nicking DNA activity varies with individual HNH endonuclease.

Most HNH endonucleases contain a Zn-finger motif, and the Zn ion is regarded as a cofactor for maintaining their overall structures. Our previous study suggests that Zn^2+^ enables GVE2 HNHE to nick DNA at high temperature^21^. Here, we found that GVE2 HNHE and the Zn ion were co-crystalized, supporting the importance of the Zn ion for DNA nicking. Ku *et al*. proposed that the zinc ion not required for DNA binding but is essential for DNA hydrolysis^31^, however, Saravanan *et al*. suggested the dual role of the zinc ion in maintaining structural integrity and inducing DNA sequence specificity of *Kpn*I restriction endonuclease^32^. Interestingly, GVE2 HNHE is able to nick DNA while the H93A, N108A and H118A mutants abolish the ability to nick DNA in the presence of Zn^2+^ (Fig. 7), suggesting that the substitutions may completely disrupt the interactions between the enzyme and Zn^2+^. However, how the Zn-finger motif in GVE2 HNHE associates with DNA remains unclear. The function of the conserved residues C76, C79, C114 and C117 in the Zn-finger domain in GVE2 HNHE needs to be clarified. Functional analysis of the Zn-finger domain in GVE2 HNHE by engineering the C76A, C79A, C114A and C117A mutants is under investigation in our laboratory. Overall, these observations suggest that Zn^2+^ is important for GVE2 HNHE to nick DNA.

Mn^2+^ and Zn^2+^ ions in *E. coli* are estimated at much lower concentration with 0.2–0.4 mM for Mn^2+^ and 0.1 mM for Zn^2+^ (cells grown in LB broth) (Bionumbers database at the web site: http://bionumbers.hms.harvard.edu)^4^. Here, we found that 0.2–0.4 mM Mn^2+^ or 0.1 mM Zn^2+^ stimulated GVE2 HNHE to completely nick DNA (Figs 6A and 7A). However, these two nicking patterns of GVE2 HNHE in the presence of Mn^2+^ or Zn^2+^ vary sharply: a wide range of Mn^2+^ concentration is favorable for nicking while only a narrow range of Zn^2+^ concentration can stimulate the enzyme to nick DNA. Comparison of the two crystal structures of GVE2 HNHE, with Mn^2+^ and Zn^2+^, provides a rational explanation for the observation. In the presence of Mn^2+^, the bond length formed between the residues C76, C79, C114 and C117 in GVE2 HNHE and Mn^2+^ became shorter than that formed in the presence of Zn^2+^. The short bond length may enable GVE2 HNHE to bind to Mn^2+^ with an enhanced affinity, which was confirmed by the Mn^2+^- and Zn^2+^-enzyme binding assays (Fig. 8). Therefore, we first provided structural and biochemical mechanistic analysis of DNA nicking by GVE2 HNHE in the presence of Mn^2+^ as the optimal cofactor. Although Mg^2+^, Mn^2+^, and Zn^2+^ ion concentrations are not known in the natural host *Geobacillus*, the nicking reaction of GVE2 HNHE could be dependent on Mg^2+^ in the host since Mg^2+^ is most likely cognate cofactor for the nicking reaction, which is supported by our previous data that Mg^2+^ can stimulate GVE2 HNHE to nick DNA^21^.

The crystal structures of several HNH endonucleases with DNA substrate have been solved^27^,^33^,^34^, which provide a wealth of information regarding mechanisms for DNA nicking. However, our attempt to solve the crystal structure of the GVE2 HNHE-DNA complex was unsuccessful. In addition, we were unable to detect binding of GVE2 HNHE to DNA through either an electrophoretic mobility shift assay or a gel filtration assay (data not shown). Thus, we proposed that the high catalytic efficiency of GVE2 HNHE may cause the interaction between the enzyme and the DNA to be undetectable because the H93A mutant cannot bind to DNA in our binding assays (data not shown).

In conclusion, our work is the first to reveal structural and biochemical mechanistic insight into DNA nicking of the thermophilic bacteriophage GVE2 HNH endonuclease. It has shown that residues H93, N109 and H118 in the conserved HNH motif in the enzyme play important roles in maintaining structural conformation and in DNA nicking. In addition, we are the first to have co-crystalized GVE2 HNHE with Mn^2+^, providing structural evidence for DNA nicking catalyzed by the enzyme in the presence of Mn^2+^ as the optimal cofactor.

## Methods

### Construction of the GVE2 HNHE N109A and H118A mutants

The GVE2 HNHE H93A mutant was constructed as described previously^21^. Similarly, the GVE2 HNHE N109A and H118A mutants were engineered using a single primer site-directed mutagenesis method, following the experimental procedure of Zhang *et al*.^35^. The mutagenic primer sequences for engineering the GVE2 HNHE N109A and H118A mutants were 5′-*CGATTAGATATGGACGCCCTGCAATCGTTATGC-3′, and 5′-*CGTTATGCCAAGCCTGCGCTAACAGAAAGACGGCGG (* indicates that C is phosphorylated and the underscored bases are mutation bases), respectively. The mutations were verified by sequencing.

### Protein expression and purification of the wild type and mutant GVE2 HNHEs

The wild type and mutant GVE2 HNHEs were over-expressed, purified, and quantified as described previously^21^. For biochemical analysis, the wild type and mutant GVE2 HNHE proteins were dialyzed in a storage buffer (50 mM Tris-HCl pH 8.0, 0.1 mM EDTA (ethylenediaminetetraacetic acid), 1 mM DTT (dithiothreitol) and 50% glycerol). For crystallization assay, the wild type GVE2 HNHE protein was dialyzed against a buffer containing 20 mM Tris-HCl pH 8.0, 50 mM NaCl, and 2 mM DTT, then concentrated to 25 mg/ml.

### Crystallization of GVE2 HNHE

Crystallization was performed by sitting-drop vapor diffusion at 293 K. The crystal of GVE2 HNHE with 10 mM Zn^2+^ (final concentration) was grown in a reservoir solution containing 0.1 M HEPES, 0.2 M sodium chloride, pH 7.5 and 25% (w/v) polyethylene glycol 4000. To obtain the crystal structure of GVE2 HNHE with Mn^2+^, 10 mM Mn^2+^ (final concentration) was added into the protein solution instead of Zn^2+^. The heavy-atom derivative crystals were obtained by soaking in 10 mM HgCl_2_ for 3 sec. All the crystals obtained were subjected to a post-crystallization procedure by dehydrating by evaporation against air for 5 min at 297 K, and then flash frozen in liquid nitrogen.

### Data collection and structure determination

Diffraction data of GVE2 HNHE with Zn^2+^, Mn^2+^ and Hg-SAD were collected at the BL17U beamline of Shanghai Synchrotron Radiation Facility, the beamline BL1A and BL5A at the Photon Factory in Japan and the beamline 1W2B at Beijing Synchrotron Radiation Facility, respectively. Data were integrated and scaled with the HKL2000 package^36^. The positions of heavy atoms and the initial phases of GVE2 HNHE were calculated by SAD using HKL2MAP^37^and Phenix^38^ with a mercury derivative data set. The structure of GVE2 HNHE with Mn^2+^ was determined by molecular replacement with the Phaser programmer in the CCP4 programed suite^39^,^40^. In addition, according to the anomalous difference Fourier maps calculated with a data set collected at a wavelength of 1.8 Å, the location of Mn^2+^ was confirmed. The structure refinements were carried out with Refmac and Phenix^38^,^41^. Model building was carried out using Coot^42^. MolProbity was used to validate the two structures^43^. A summary of data collection and final refinement statistics are listed in Table 1.The program Pymol (http://www.pymol.org) was used to prepare the crystal structure figures of GVE2 HNHE. The sequence alignment figure (Fig. 1A) was prepared using ESPript^44^.

### Accession numbers

The atomic coordinates and structure factors have been deposited in the PDB database under accession codes 5H0M and 5H0O.

### Circular dichroism measurements

The wild type GVE2 HNHE and its mutants harboring the H93A, N109A, and H118A substitutions were dialyzed into 50 mM PBS (phosphate-buffered saline) pH7.5 for CD analysis. The CD spectra were recorded at 20 °C from 200 to 250 nm using a J-810 spectropolarimeter and a cuvette of path length 0.2 cm. The proteins were at a concentration of 1.5 mg/ml. The spectra were collected at a scanning rate of 50 nm/min, and triplicate spectrum readings were collected per sample. The CD spectral data were reported as mean residue ellipticity [θ], and the CD wavelength spectra were smoothed as described by Savitsky and Golay^45^.

Thermal stability of the wild type and mutant GVE2 HNHEs was examined by the following changes in the spectrum in CD analysis with increasing temperature (30–90 °C). The proteins were at a concentration of 1.5 mg/ml. A single wavelength (222 nm) was selected to monitor the GVE2 HNHE protein structure, and the signal at that wavelength was recorded continuously as the temperature was increased. The CD wavelength spectra were smoothed using Origin software.

CD spectra for monitoring Mn^2+^- or Zn^2+^-dependent structural changes in GVE2 HNHE were recorded after incubation of the enzyme with different concentrations (2 nM, 10 nM, 50 nM, 200 nM, 1 mM and 5 mM) of Mn^2+^ or Zn^2+^ on ice for 5 min. Binding constants were determined from the plot of ellipticity changes at a wavelength of 222 nm with an increasing metal ion concentration. Apparent dissociation constants (*K*_d_) were determined by fitting the curves to Hill analysis using Origin software.

### DNA nicking assays

DNA nicking assays were carried out in the reactions (20 μl) containing 200 ng the plasmid pET-30a DNA, 20 mM Tris-HCl pH 8.0, 1 mM DTT, GVE2 HNHE or its mutant (H93A, N108A or H119A) at various concentrations (5, 20, 50, 100, 200 or 400 nM), 2 mM MnCl_2_, 0.1 mg/ml BSA (bovine serum albumin) and 10% glycerol. The reactions were performed at 60 °C for 15 min. The reactions were terminated with the addition of EDTA at a final concentration of 100 mM and 1 × DNA loading buffer (New England Biolabs). The nicking product was analyzed by electrophoresis through 1% agarose gels.

## Additional Information

**How to cite this article:** Zhang, L. *et al*. Structural and functional characterization of deep-sea thermophilic bacteriophage GVE2 HNH endonuclease. *Sci. Rep.* **7**, 42542; doi: 10.1038/srep42542 (2017).

**Publisher's note:** Springer Nature remains neutral with regard to jurisdictional claims in published maps and institutional affiliations.

## Figures and Tables

**Table 1:** Data collection and refinement statistics.

	GVE2 HNHE-Zn^2+^		GVE2 HNHE-Mn^2+^	Hg-SAD
**Data collection**				
Wavelength (Å)	0.98		1.1	0.98
Space group	P4_3_2_1_2		P4_3_2_1_2	P4_3_2_1_2
Cell dimensions				
* a, b, c* (Å)	66.3, 66.3,51.5	66.9, 66.9,51.5		65.2, 65.2,50.4
* α, β, γ* (°)	90.0, 90.0, 90.0	90.0, 90.0, 90.0		90.0, 90.0, 90.0
Resolution (Å)	50–1.52 (1.55–1.52)^a^	50–1.53 (1.55–1.53)^a^		50–1.80 (1.84–1.80)^a^
*R*_merge_	0.066 (0.541)	0.065 (0.993)		0.070 (0.628)
*I*/σ*I*	65.2 (6.7)	21.6 (2.1)		28.5 (3.0)
Completeness (%)	99.7 (100)	99.8 (100)		99.4 (98.8)
Redundancy	13.7	13.4		15.9
**Refinement**				
Resolution (Å)	50–1.52	50–1.53		
No. reflections	17,215	18,142		
*R*_work/_*R*_free_	0.144/0.168	0.141/0.174		
No. atoms				
* *Protein	780	780		
Metal ion	1 Zn^2+^	1 Mn^2+^		
* *Water	146	190		
B-factors	22.2	21.7		
R.m.s deviations				
* *Bond lengths (Å)	0.007	0.008		
* *Bond angles (°)	1.13	0.96		
Ramachandran Plot analysis Favoured (%)	100	98.9		
Outliners (%)	0	0		
PDB code	5H0M	5H0O		

^a^The values in parenthesis mean those of the highest resolution shell.
